# Inappropriate use of antibiotics and its predictors in pediatric patients admitted at the Central Hospital of Nampula, Mozambique

**DOI:** 10.1186/s13756-022-01115-w

**Published:** 2022-06-02

**Authors:** Sancho Pedro Xavier, Audêncio Victor, Graciano Cumaquela, Melsequisete Daniel Vasco, Osiyallê Akanni Silva Rodrigues

**Affiliations:** 1 Departamento de Farmácia, Faculdade de Ciências de Saúde, Universidade Lúrio, Bairro do Marrere, R. no4250, Nampula, Mozambique; 2 Instituto de Saúde Coletiva, Universidade Federal da Bahia, Rua Basílio da Gama, S/nº Campus Universitário Canela, Salvador, BA 40110-040 Brazil

**Keywords:** Antibiotic prescription, Inappropriate use of antibiotics, Pediatrics

## Abstract

**Background:**

Antibiotics are synthetic or natural substances used to treat bacterial infectious diseases. When used incorrectly, they can be a factor in the development of antimicrobial resistance, increased treatment time, costs, and mortality. The present study aimed to assess the pattern of inappropriate use of antibiotics and their predictors in pediatric patients admitted to the Central Hospital in Nampula, Mozambique.

**Methods:**

A cross-sectional, retrospective study with a quantitative approach was conducted between January and July 2019. The population consisted of children ages 0–10 years hospitalized in the pediatric ward I. Binary logistic regression was used to determine risk factors for the inappropriate use of antibiotics with 95% confidence interval.

**Results:**

The prevalence of antibiotic use among pediatric patients was 97.5%. Of the 464 antibiotics prescribed, 39.9% were for patients suffering from gastroenteritis, 21.8% and 9.1% for those affected with pneumonia and malaria, respectively. Most antibiotics were for parenteral use (95.9%, 445/464). Many (36.5%) of the prescriptions had errors, primarily in the duration of treatment (74.0%) or dosage (24.4%). Binary logistic regression analysis revealed that patients prescribed ≥ 3 antibiotics (OR = 2.83, 95% CI 1.245–6.462, *p*-value = 0.013) or hospitalized for a short time (OR = 1.88, 95% CI 1.133–2.3140, *p*-value = 0.015) were more likely to experience inappropriate use of antibiotics.

**Conclusion:**

The study showed both a high prevalence of antibiotic use and a high error rate in prescriptions, especially among patients prescribed ≥ 3 antibiotics or hospitalized for a short time. These results are concerning, since inappropriate and excessive use of antibiotics is a major factor in the development of antibiotic-resistant microorganisms. Therefore, policies to reduce the inappropriate and excessive use of antibiotics are necessary.

## Background

Antibiotics are drugs of natural or synthetic origin used to treat infectious diseases, with the ability to destroy or inhibit the proliferation of microorganisms. They are the most-used drugs in pediatric patients and play a vital role in the treatment of infectious diseases worldwide [1]. Studies have shown that about 86% of hospitalized children received at least one antibiotic [2]. Currently, these drugs are used excessively and inappropriately, resulting in risks to the user, such as ineffective treatment and the development of microorganism resistance [3]. The worldwide excessive use of antibiotics in agriculture and human medicine results in the proliferation and dissemination of a multitude of antibiotic-resistant genes, which is one of the most serious global public health threats [4]. This practice of excessive and inappropriate use of antibiotics is the main global cause of bacterial resistance [3]. The inappropriate use of antibiotics includes errors in prescription, administration, dosage and duplication of the same product [5]. Some studies have shown that the percentage of improper use of some antibiotics in hospitals ranges from 20 to 80% [6, 7]. Moreover, patients with antibiotic-resistant infections are more likely to experience ineffective treatment, recurrent infection, delayed recovery, and even death [3]. Therefore, about 6.5% of morbidity and mortality in hospitalizations is associated with inappropriate antibiotic prescription [8]. In the United States and Canada, 30–50% of antibiotics are used incorrectly. Similarly, in some Asian and African nations, 50% of prescribed antibiotics are identified as inappropriate [9, 10]. According to the World Health Organization (WHO), antimicrobial resistance among pathogens responsible for common infections is alarmingly high [11]. In Middle East regions, 90% of hospitalized newborns with sepsis had bacterial resistance to antibiotics [12]. In sub-Saharan Africa, 66% of cases of neonatal sepsis and meningitis are caused by antibiotic-resistant bacteria [13]. A current, high-level report estimates that by 2050, “10 million people will die every year due to antimicrobial resistance (AMR) unless a global response to the problem of AMR is mounted” [14]. Reducing misuse is essential to reducing not only antibiotic resistance but adverse reactions as well. Therefore, proper prescription is an essential strategy to reduce the inappropriate and excessive use of antibiotics, limiting them to careful application in certain circumstances, in order to prevent the rapid growth of bacterial resistance to antibiotics. Knowledge about the patterns of incorrect use of antibiotics and their risk factors in the pediatric population is vital for the creation of programs, policies, and actions related to the dispensing of these drugs in this population. Thus, the present study aimed to assess the pattern of inappropriate use of antibiotics and their predictors in pediatric patients admitted to the Central Hospital in Nampula, Mozambique.

## Methods

### Study design

A cross-sectional, retrospective study with a quantitative approach was conducted in patients hospitalized at the Central Hospital of Nampula, Mozambique. Pediatrics I is a ward that treats patients with various pathologies, categorized into gastrointestinal, respiratory, and general diseases. The survey was carried out in the year 2020.

### Population and sample size

From January to July 2019, a total of 1745 patients between the ages of 1 and 120 months were hospitalized in that ward. The minimum sample size for the study was 315, estimated through the single population proportion formula. To obtain a representative sample, the simple randomization technique was used, in which each element of the population has an equal chance of being selected to be part of the sample.

### Data collection

Data were collected through supervision of the principal investigator. A data collection instrument was used containing the patients' sociodemographic and clinical characteristics and information concerning the antibiotics used during hospital stay. The enumerated record book of patients admitted to the ward was used to obtain the master list of the population for randomization, from which the research participants were selected. When a clinical file was found to lack information important to the study, such as age, sex, weight, medical diagnosis, outcome, dosage, route of administration, duration of treatment, and length of hospitalization, that participant was excluded and was substituted with a different element selected at random. No information was found in all medical records regarding microbiological reasons for prescribing antibiotics for clinical conditions.

### Study variables

The proper or improper use of antibiotics was considered the dependent variable and considered as the study outcome variable, in which it was classified as dichotomous categorial. It was coded using code one (1) for misuse, which is inappropriate antibiotic use, and zero (0) for correct use, which is proper use. The variable of interest was code 1. The independent variables were sex, age, weight, medical diagnosis, outcome (discharge, Abandonment, or death), prescribed antibiotics, dosage, route of administration, duration of treatment, and length of hospitalization; and each was classified as numerical or categorical. The numerical variables were age, weight, number of antibiotics prescribed and duration of treatment. The categorical variables were sex, medical diagnosis, outcome, dosage, dose, route of administration and length of hospitalization. The age variable was also considered as categorical: infant (28 days–12 months), toddler (1–2 years), preschool (3–5 years), and school (6–10 years). The weight variable was transformed into categorical: < 10, 10–18.9, and ≥ 19. The dosage was categorized into underdose, overdose, and correct dose. Duration of treatment was transformed into categorical, according to Table 1. The variables used to derive the outcome of interest were age; weight; length of hospital stay (short, < 5 days; long, ≥ 5), which was determined according to Iftikhar et al. [15]; and number of antibiotics prescribed.

**Table 1:** Demographic and clinical characteristics of pediatric patients hospitalized in the Pediatrics I ward

Variables	(n = 315)	%
*Sex*		
Male	199	63.2
Female	116	36.8
*Age*		
[1–2 years]	162	51.4
[28 days–12 months]	74	23.5
[3–5 years]	41	13.0
[6–10 years	38	12.1
*Age in months*		**(**mean ± SD) = 30.44 ± 26.07
*Weight* (kg)		
< 10	154	48.9
10–18.9	136	43.2
≥ 19	25	7.9
*Number of prescribed antibiotics*		
1	187	60.9
2	90	29.3
≥ 3	30	9.7
*Length of hospitalization* (days)		
Short [average = 2.85, range 0–4]	194	61.6
Long [average = 8.99, range 5–39]	121	38.4
*Duration of antibiotic therapy* (days)		
0–5	382	82.3
6–8	40	8.6
9–14	22	4.7
≥ 15	20	4.3
*Outcome*		
Discharge	301	95.6
Abandonment	8	2.5
Death	6	1.9

### Determining prescription errors

The antibiotics were considered appropriately used if the dosage written on the prescription was in accordance with the patient’s weight. Antibiotics were considered inappropriately used in five cases: (1) combination of antibiotics of the same class (duplication); (2) wrong antibiotic selected for the disease; (3) non-recommended route of administration; (4) wrong duration of antibiotic use; and (5) dosage error, in which the prescribed dose was higher or lower and short or long duration of treatment than recommended by international guidelines and databases. The dosage error was determined after assessing the 10% deviation from the lower and upper limits of normal dosing range. Prescription doses with an error above 10% were considered overdoses, while prescribed doses below 10% were considered underdoses. References used were the WHO guidelines [16], British National Formulary for Children (BNFC) September 2019–2020, and National Institute for Health and Care Excellence (NICE) [17, 18]. These references were used because of the wide details on dose, dosage, duration of treatment and indication.

### Data analysis

Data were entered into SPSS 25.0 software for cleaning and analysis. Microsoft Excel was used for graphic creation. Descriptive statistics was applied to the sociodemographic, clinical data and information of antibiotics used in terms of frequencies and proportions, measures of central tendency (mean, median, minimum and maximum values) and dispersion (standard deviation). The multivariate logistic regression model was applied to identify the predictor variables for inappropriate antibiotic use after selecting the variables for the model, thus, the Odds Ratio (OR) was applied to determine the likelihood of inappropriate use of antibiotics occurring with 95% confidence interval (CI) and *p*-value < 0.05 for statistically significant differences between the independent variables with the outcome of interest. To verify that the regression model was generalizable and the correlation levels appropriate, the multicollinearity analysis between the independent variables was performed.

## Results

### Demographic and clinical features of patients

Of the 315 pediatric patients selected for the study, 63.2% were male. The majority (162; 51.4%) were between 1 and 3 years of age. The mean age in months was 30.44 (SD ± 26.07) and the median was 21, with 5 months as the minimum age and 120 months the maximum. Nearly half of the pediatric patients (48.9%) weighed less than 10 kg. As to the length of hospital stay (in days), 61.6% had a short hospital stay, with an average of 2.85 days; and 39.4% had a long hospital stay, with an average of 8.99 days (Table 1).

### Description of used antibiotics

Of the 315 pediatric hospitalized patients, 307 were prescribed antibiotics. A total of 464 prescriptions of antibiotics were made. The prevalence of antibiotic use during the study period was 97.5% (307/315). Many antibiotics (39.9%) were prescribed for patients suffering from gastroenteritis, 21.8% for those with pneumonia, and 9.1% for patients affected with malaria (Table 2). Most antibiotics prescribed (443/464; 95.5%) were for parenteral use, followed by oral use (19; 4.1%). As for oral antibiotics, 84.2% (16/19) were in suspension form and 15.8% (3/19) tablets.

**Table 2:** Distribution of the antibiotic prescriptions by diagnosis among patients

Diagnosis (ICD-10)	(n = 464)	%	Diagnosis (ICD-10)	n	%
Gastroenteritis (A09, K52)	185	39.9	Epilepsy (G40.0)	6	1.3
Bronchopneumonia (J18.0)	101	21.8	Meningoencephalitis (G04)	5	1.1
Malaria (B50)	42	9.1	Pyoderma (L0.80)	4	0.9
Febrile convulsions (R59.0)	34	7.3	Cerebral palsy (G80)	4	0.9
Sepsis (A41.9)	16	3.4	Heart disease (I25-I69)	4	0.9
Anemia (D53)	12	2.6	Haemangioma (T18)	3	0.6
Typhoid fever (A01.))	11	2.4	Neoplasm (D36, C80.9)	2	0.4
Asthma (J46)	7	1.5	Injury (T14.9)	2	0.4
Hydrocephalitis (G90)	6	1.3	Allergy (T78.4)	2	0.4
Pharyngotonsillitis (J06.8)	7	1.5	Pains (R52)	1	0.2
Pharyngitis (J02, J31.2)	3	0.6	Splenomegaly (R16, B54)	1	0.2
Tonsillitis (J03)	2	0.4	Laryngitis (J04, J37)	1	0.2
Dermatitis (L20-L30)	2	0.4	Burn (T29)	1	0.2

**Fig. 1:**
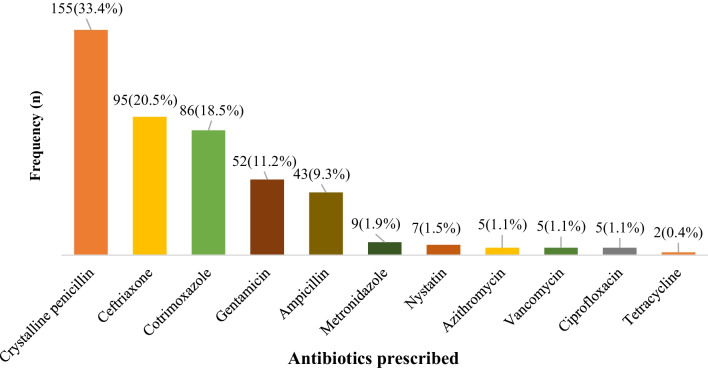
Antibiotics prescribed for hospitalized pediatric patients. *Note: Crystalline penicillin (inj.), Ceftriaxone (inj.), Cotrimoxazole (both inj. and oral), Gentamicin (inj.), Ampicillin (inj.), Metronidazole (both inj. and oral), Nystatin (oral), Azithromycin (both inj. and oral), Vancomycin (inj.), Ciprofloxacin (both inj. and oral) and Tetracycline (unguentum)

Figure 1 shows that the most-used antibiotics were crystalline penicillin (155/464, 33.4%), ceftriaxone (95/464, 20.5%), co-trimoxazole (86/464, 18.5%), gentamicin (52/464, 11.2%), and ampicillin (43/464, 9.3%).

### Description of antibiotic use errors

A total of 36.5% (112/307) of antibiotic prescriptions had errors; thus, 34.9% (162/464) of individual antibiotics were incorrectly prescribed. 57.1% (64/112) of prescriptions had one error, while 24.1% (27/112), 8.0% (9/112), and 10.7% (12/112) had respectively two, three, and four errors. An error rate of 100% was detected in patients suffering from hydrocephalitis (6/6), meningo encephalitis (5/5), haemangioma (3/3) and pharyngitis (3/3), with the next highest rates in sepsis (63%; 10/16), pharygotonsillitis (57%; 4/7) and typhoid fever (55%; 6/11). The errors found were duration of treatment (74.1%; 143/193), dosage (24.4%; 47/193), and duplication of therapy (1.6%; 3/193). Dosage errors were 53.2% (25/47) overdoses and 46.8% (22/47) were underdoses, while duration errors were 76.2% (109/143) too short and 23.8% (34/143) too long.

### Analysis to determine risk factors for inappropriate use of antibiotics

Binary logistic regression was applied to verify whether age, weight, number of antibiotics, and length of hospital stay predict inappropriate use of antibiotics. The regression model containing age, number of antibiotics, and length of hospital stay was significant (X2 (1) = 15.005; *p* < 0.020; R2 Cox & Snell = 0.48). The chance of antibiotics being used inappropriately was almost three times greater in pediatric patients with a prescription of ≥ 3 antibiotics (OR = 2.83, 95% CI 1.245–6.462, *p*-value = 0.013), a nearly 200% increase in relation to patients prescribed only one antibiotic. In patients hospitalized for a short time, the chance of antibiotics being used inappropriately was 1.8 times higher (OR = 1.88, 95% CI 1.133–3.140, *p*-value = 0.015), a 100% increase in odds compared to patients hospitalized for a long period (Table 3).

**Table 3:** Determination of risk factors for inappropriate use of antibiotics

Variables	Clinical predictors	OR	(95% CI)	*p* value
Age	1–2 years	–		
	28 days–12 months	0.75	0.382–1.482	0.410
	3–5 years	1.16	0.526–2.574	0.708
	6–10 years	1.20	0.432–3.332	0.728
Weight (kg)	< 10	–		
	10–18.9	0.96	0.518–1.813	0.921
	≥ 19	1.70	0.466–6,216	0.421
Number of antibiotics per prescription	1	–		
	2	1.40	0.819–2.406	0.217
	≥ 3	2.83	1.245–6.462	0.013*
Hospitalization time (days)	Short (< 4)	1.88	1.133–3.140	0.015*
	Long (≥ 5)	–		

^*^Shown to have statistical significance with *p*-value less than or equal to 0.05 (95% CI)

## Discussion

Antibiotics are the most commonly prescribed type of drug, and their use in the pediatric population is a huge concern in some countries. Today, antibiotic resistance is one of the most serious public health problems that has arisen from excessive and inappropriate antibiotic use in both humans and animals [19].

Studies have shown that antibiotics are overused among pediatric patients. The 97.5% prevalence rate discovered in the present study converges with rates found by Abubakar in a hospital in Nigeria (80.1%) and by Monteiro LGS et al. in two hospitals (Central and General) in Maputo (97.6%) [20, 21]. Lower prevalence (43.5%) was found by Wai et al., in a tertiary hospital in Malaysia [22]. All these antibiotic prescription rates are alarmingly higher than the WHO-recommended frequency of 20.0–26.8% in hospitals [23], possibly because the health systems in these countries lack resources for more appropriate medications. The danger is that when antibiotics are used unnecessarily and excessively, they provide a means for the development of antibiotic-resistant microorganisms, which can lead to ineffective treatment, recurring infections, increased treatment costs, or even mortality. In the present study, gastroenteritis (39.9%), pneumonia (21.8%), and malaria (9.1%) were the diagnoses for which antibiotics were most commonly prescribed. Chaw et al., in their research, similarly evidenced that antibiotics were prescribed to pediatric inpatients at a Gambian hospital most frequently for pneumonia (37.1%), followed by sepsis (14.1%) [24]. Monteiro LGS et al., identified that antibiotics were prescribed for 100% of patients suffering from pneumonia, fevers, sepsis, and gastroenteritis, and for 97.8% of patients suffering from malaria [21]. The present study observed that crystallized penicillin (33.4%), ceftriaxone (20.5%), cotrimoxazole (18.5%), and gentamicin (11.2%) were the most-used antibiotics. Muslim and Meinisasti found a divergent rates of gentamicin (34.9%) and ampicillin (34.3%) [25]. Labi et al., found a higher rate of use of ceftriaxone (80.5%), followed by of gentamicin (76.5%), while Chaw et al. identified ampicillin (19.5%), followed by gentamicin (14.5%) and ceftriaxone (12.8%) as the most common antibiotics used [26, 27]. The present research reveals that parenteral antibiotics represent the most-used (95.5%) dosage form. Labi et al. found a convergent rate of 83.5% [27]. Monteiro LGS et al. also found that the most-used antibiotics were parenteral, but with a much lower frequency (52.9%) [21], while Mgbahurike et al. observed a very high rate of oral antibiotics (86.3%) [28]. This high use of parenteral antibiotics found in the present study is far above the WHO-recommended frequency of 13.4–24.1% injectable [23], and may be due to the age of the patients and the severity of their illnesses. Also, this may be due resource-poor setting (shortage of routine microbiological cultures, lack of workers, equipment and financial resources) which may result in empirical use and poor availability of antibiotics [29, 30].

As to the nature of the errors, the present research observed 36.5% of medical prescriptions containing errors, with a total of 34/9% of antibiotics being improper prescribed. Iftikhar et al. [15], and Denny et al. [31], observed convergent rates of 40.8% and 22.9% improper prescriptions, respectively. A survey by Santander et al. found a similar rate of 51.9% of improper medical prescriptions [32], while Okello et al. found a rate of 68.4% [33]. In the present research, 57.1%, 24.1%, 10.7%, and 8.0% of the prescriptions presented one, two, four, and three errors, respectively. Iftikhar et al. found that 47.2% of prescriptions had one error, while 21.7% and 30.9% respectively had two and three or more errors [15]. Most of the errors detected in the present research were related to dosage (24.4%) and duration of treatment (74.1%). Fekadu et al. also observed dosage (underdose = 27.1%, overdose = 7.03%), frequency (low = 20.54%, high = 3.78%), and duration of use (short duration = 13.51%, prolonged = 0.54%) were the most common errors [34]. Oguz et al. found that 55.69% of antibiotics had errors, including frequency, (25.88%), route of administration (16.08%), and dosage (2.67%) [35]. These results are alarming, since the inappropriate use of these drugs constitutes the primary means for the emergence, growth, and dissemination of resistant microorganisms. The widespread inappropriate use of antibiotics may be due to carelessness or poor judgment of prescribers regarding the proper prescription of these drugs, considering their guidelines and patient characteristics. Also, this may be due to the use of antibiotics empirically and low availability of antibiotics in hospitals [30].

As to risk factors for the inappropriate use of antibiotics, the study showed that patients prescribed ≥ 3 antibiotics are more likely (about 3 times more) to have some inappropriate use of antibiotics than those prescribed only one antibiotic, and that patients with a short hospital stay have about twice the chance of experiencing some inappropriate use of antibiotics than those who stayed ≥ 5 days. Iftikhar et al. similarly found that prescribing three or more antibiotics (OR = 1.7, 95% CI 1.1–2.1, *p*-value = 0.020) is a risk factor for inappropriate antibiotic use compared to patients prescribed one antibiotic; but in contrast to the present study, those authors found that a long hospital stay (OR = 12.5, 95% CI 10.1–17.6, *p*-value < 0.001) constitutes a risk factor for experiencing some incorrect use of antibiotics [15]. Prescribers may recommend three or more antibiotics due to uncertainty of whether an infection is viral or bacterial or due to non-compliance with guidelines for antibiotic prescription. Meanwhile, pressure exerted on physicians by family members and guardians of pediatric patients may be a reason for their short hospital stay. Because inappropriate antibiotic use is a major factor in the development of resistant microorganisms, age-sensitive interventions are necessary to reduce their use in pediatrics. These policies governing the selection of the best therapeutic regimen, dose, duration of treatment, and route of administration would improve clinical outcomes from antibiotic use, reduce adverse effects such as toxicity, reduce costs, and limit the development of resistant microorganisms.

Limitations to the current study include a lack of some clinical data regarding the patients' discharge, whether it was due to a clinical improvement or to continue the therapy in an ambulatory care. Nevertheless, most patients were discharged due to clinical improvement.

## Conclusion

Antibiotic use among hospitalized pediatric patients was highly prevalent. Many prescriptions were inappropriate, with dosage and duration of treatment as the most frequent errors. Patients who were prescribed with three or more antibiotics per prescription, and had short Hospitalization time were more likely to experience prescribing errors. These results are concerning, as overuse and inappropriate use of antibiotics are major factors in the development of antibiotic-resistant microorganisms. Prioritizing the design and development of new antibacterial agents is not a good strategy to solve this worldwide health crisis; instead, the creation or improvement of policies, programs, and methods for the use of existing drugs must be the priority. Multiple interventions, such as lectures and poster distributions, are essential for educating both health professionals and the general public about the appropriate use of antibiotics. Institutions must also have up-to-date, internal forms and guidelines on the most commonly used antibiotics, to assist in decision-making on antibiotic use according to the needs of each individual patient.

## Data Availability

The datasets used and/or analyzed during the current study are available from the corresponding author on reasonable request.

## Abbreviations

AMR: Antimicrobial resistance
BNF: British National Formulary
CI: Confidence interval
IBM: International Business Machines Corporation
NICE: National Institute for Health and Care Excellence
OR: Odds ratio
SPSS: Statistical Package for the Social Sciences
SD: Standard deviation
WHO: World Health Organization

## References

[1] Moser C, Lerche CJ, Thomsen K, Hartvig T, Schierbeck J, Jensen PØ (2019). Antibiotic therapy as personalized medicine—general considerations and complicating factors. APMIS.

[2] Kebede HK, Gesesew HA, Woldehaimanot TE, Goro KK (2017). Antimicrobial use in paediatric patients in a teaching hospital in Ethiopia. PLoS ONE.

[3] Llor C, Bjerrum L (2014). Antimicrobial resistance: risk associated with antibiotic overuse and initiatives to reduce the problem. Ther Adv Drug Saf.

[4] Tyrrell C, Burgess CM, Brennan FP, Walsh F (2019). Antibiotic resistance in grass and soil. Biochem Soc Trans.

[5] Umar LW, Isah A, Musa S, Umar B (2018). Prescribing pattern and antibiotic use for hospitalized children in a Northern Nigerian Teaching Hospital. Ann Afr Med.

[6] Mama M, Mamo A, Usman H, Hussen A, Morka G (2020). Inappropriate antibiotic use among inpatients attending Madda Walabu University Goba Referral Hospital, Southeast Ethiopia: implication for future use. Infect Drug Resist.

[7] Saleem Z, Saeed H, Hassali MA, Godman B, Asif U, Yousaf M (2019). Pattern of inappropriate antibiotic use among hospitalized patients in Pakistan: a longitudinal surveillance and implications. Antimicrob Resist Infect Control.

[8] Aidara-Kane A, Angulo FJ, Conly JM, Minato Y, Silbergeld EK, McEwen SA (2018). World Health Organization (WHO) guidelines on use of medically important antimicrobials in food-producing animals. Antimicrob Resist Infect Control.

[9] McCullough AR, Pollack AJ, Hansen MP, Glasziou PP, Looke DFM, Britt HC (2017). Antibiotics for acute respiratory infections in general practice: comparison of prescribing rates with guideline recommendations. Med J Aust.

[10] Fleming-dutra KE, Hersh AL, Shapiro DJ, Bartoces M, Enns EA (2020). Prevalence of inappropriate antibiotic prescriptions among US ambulatory care visits, 2010–2011. JAMA.

[11] Prestinaci F, Pezzotti P, Pantosti A (2015). Antimicrobial resistance: a global multifaceted phenomenon. Pathog Glob Health.

[12] Le DK, Barker CIS, Irwin A, Sharland M (2014). Improving antibiotic prescribing for children in the resource-poor setting. Br J Clin Pharmacol.

[13] Okomo U, Akpalu ENK, Doare K Le, Roca A, Cousens S, Jarde A, et al. Aetiology of invasive bacterial infection and antimicrobial resistance in neonates in sub-Saharan Africa: a systematic review and meta-analysis in line with the STROBE-NI reporting guidelines. 2019;3099(19):1–16. 10.1016/S1473-3099(19)30414-1. 10.1016/S1473-3099(19)30414-1 31522858

[14] de Kraker MEA, Stewardson AJ, Harbarth S (2016). Will 10 million people die a year due to antimicrobial resistance by 2050?. PLoS Med.

[15] Iftikhar S, Sarwar MR, Saqib A, Sarfraz M, Shoaib Q-U-A (2019). Antibiotic prescribing practices and errors among hospitalized pediatric patients suffering from acute respiratory tract infections: a multicenter, cross-sectional study in Pakistan. Medicina (Kaunas).

[16] Lutsar I. WHO report on consensus guidance on pediatric dosing regimens for access antibiotics on the essential medicine list for children.

[17] National Institute for Health and Care Excellence (NICE) and Public Health England (PHE). Summary of antimicrobial prescribing guidance-managing common infections. 2019.

[18] National Institute for Health and Care Excellence Guideline. Pneumonia (community-acquired): antimicrobial prescribing. 2019.

[19] Huttner A, Harbarth S, Carlet J, Cosgrove S, Goossens H, Holmes A (2013). Antimicrobial resistance: a global view from the 2013 World Healthcare-Associated Infections Forum. Antimicrob Resist Infect Control.

[20] Abubakar U (2020). Antibiotic use among hospitalized patients in northern Nigeria: a multicenter point-prevalence survey. BMC Infect Dis.

[21] Monteiro LGS, Chaúque A, Barros MP, Irá TR (2017). Determinants of antibiotic prescription in paediatric patients: the case of two hospitals in Maputo, Mozambique. S Afr J Child Health.

[22] Wai D, Tham J, Abubakar U, Tangiisuran B (2020). Prevalence and predictors of antibiotic use among children visiting the Emergency Department in a Tertiary Hospital in Malaysia. Eur J Pediatr.

[23] Bilal AI, Osman ED, Mulugeta A (2016). Assessment of medicines use pattern using World Health Organization ’ s Prescribing, Patient Care and Health facility indicators in selected health facilities in eastern Ethiopia. BMC Health Serv Res.

[24] Chaw PS, Schlinkmann KM, Raupach-rosin H, Karch A, Pletz MW, Huebner J (2018). Antibiotic use on paediatric inpatients in a teaching hospital in the Gambia, a retrospective study. Antimicrob Resist Infect Control.

[25] Muslim Z, Meinisasti R (2016). Short communications rationality of antibiotic usage in paediatrics in Bengkulu, Indonesia: Gyssens’ criteria and type of therapy analysis. Indian J Pharm Sci.

[26] Chaw PS, Schlinkmann KM, Raupach-Rosin H, Karch A, Pletz MW, Huebner J (2018). Antibiotic use on paediatric inpatients in a teaching hospital in the Gambia, a retrospective study. Antimicrob Resist Infect Control.

[27] Labi A, Obeng-nkrumah N, Sunkwa-mills G, Bediako-bowan A, Akufo C, Bjerrum S (2018). Antibiotic prescribing in paediatric inpatients in Ghana: a multi-centre point prevalence survey. BMC Pediatr.

[28] Mgbahurike AA, Ojiyi ID, Chijioke-Nwauche IN (2020). Antibiotic utilization pattern in pediatrics unit south–south of Nigerian Teaching Hospital. J Med Biomed Appl Sci.

[29] Allwell-Brown G, Hussain-Alkhateeb L, Kitutu FE, Strömdahl S, Mårtensson A, Johansson EW (2020). Trends in reported antibiotic use among children under 5 years of age with fever, diarrhoea, or cough with fast or difficult breathing across low-income and middle-income countries in 2005–17: a systematic analysis of 132 national surveys from 73 countri. Lancet Glob Health.

[30] Sartelli M, C Hardcastle T, Catena F, Chichom-Mefire A, Coccolini F, Dhingra S, et al. Antibiotic use in low and middle-income countries and the challenges of antimicrobial resistance in surgery. Antibiot (Basel, Switzerland). 2020;9(8):497. 10.3390/antibiotics9080497 PMC7459633 32784880

[31] Denny KJ, Gartside JG, Alcorn K, Cross JW, Maloney S, Keijzers G (2019). Appropriateness of antibiotic prescribing in the Emergency Department. J Antimicrob Chemother.

[32] Santander BC, Alonso EC, Carrión AS, Fuentes LM, Flores ID, Vargas JC, et al. Adecuación de la prescripción de antimicrobianos en población pediátrica en un servicio de urgencias hospitalario. In: Anales de Pediatría. Elsevier; 2018. p. 259–65. 10.1016/j.anpedi.2017.06.001. 10.1016/j.anpedi.2017.06.001 28711429

[33] Okello N, Oloro J, Kyakwera C, Kumbakumba E, Obua C (2020). Antibiotic prescription practices among prescribers for children under five at public health centers III and IV in Mbarara district. PLoS ONE.

[34] Fekadu G, Abdisa E, Fanta K (2019). Medication prescribing errors among hospitalized pediatric patients at Nekemte Referral Hospital, western Ethiopia: cross-sectional study. BMC Res Notes.

[35] Oğuz E, Bebitoğlu BT, Nuhoğlu Ç, Çağ Y, Hodzic A, Temel F (2021). Evaluation of antibiotic use among hospitalised patients in a paediatric department of a training hospital in Turkey. Int J Clin Pract.

